# Effects of S1 Cleavage on the Structure, Surface Export, and Signaling Activity of Human Notch1 and Notch2

**DOI:** 10.1371/journal.pone.0006613

**Published:** 2009-08-24

**Authors:** Wendy R. Gordon, Didem Vardar-Ulu, Sarah L'Heureux, Todd Ashworth, Michael J. Malecki, Cheryll Sanchez-Irizarry, Debbie G. McArthur, Gavin Histen, Jennifer L. Mitchell, Jon C. Aster, Stephen C. Blacklow

**Affiliations:** 1 Department of Pathology, Brigham and Women's Hospital and Harvard Medical School, Boston, Massachusetts, United States of America; 2 Department of Chemistry, Wellesley College, Wellesley, Massachusetts, United States of America; University of Texas MD Anderson Cancer Center, United States of America

## Abstract

**Background:**

Notch receptors are normally cleaved during maturation by a furin-like protease at an extracellular site termed S1, creating a heterodimer of non-covalently associated subunits. The S1 site lies within a key negative regulatory region (NRR) of the receptor, which contains three highly conserved Lin12/Notch repeats and a heterodimerization domain (HD) that interact to prevent premature signaling in the absence of ligands. Because the role of S1 cleavage in Notch signaling remains unresolved, we investigated the effect of S1 cleavage on the structure, surface trafficking and ligand-mediated activation of human Notch1 and Notch2, as well as on ligand-independent activation of Notch1 by mutations found in human leukemia.

**Principal Findings:**

The X-ray structure of the Notch1 NRR after furin cleavage shows little change when compared with that of an engineered Notch1 NRR lacking the S1-cleavage loop. Likewise, NMR studies of the Notch2 HD domain show that the loop containing the S1 site can be removed or cleaved without causing a substantial change in its structure. However, Notch1 and Notch2 receptors engineered to resist S1 cleavage exhibit unexpected differences in surface delivery and signaling competence: S1-resistant Notch1 receptors exhibit decreased, but detectable, surface expression and ligand-mediated receptor activation, whereas S1-resistant Notch2 receptors are fully competent for cell surface delivery and for activation by ligands. Variable dependence on S1 cleavage also extends to T-ALL-associated NRR mutations, as common class 1 mutations display variable decrements in ligand-independent activation when introduced into furin-resistant receptors, whereas a class 2 mutation exhibits increased signaling activity.

**Conclusions/Significance:**

S1 cleavage has distinct effects on the surface expression of Notch1 and Notch2, but is not generally required for physiologic or pathophysiologic activation of Notch proteins. These findings are consistent with models for receptor activation in which ligand-binding or T-ALL-associated mutations lead to conformational changes of the NRR that permit metalloprotease cleavage.

## Introduction

Notch proteins are modular, single-pass transmembrane receptors that transduce signals between neighboring cells in multicellular organisms. Binding of ligands to Notch receptors triggers a proteolytic cascade that releases the intracellular part of the receptor from the membrane, allowing it to move into the nucleus where it induces transcription of target genes. Notch signals have highly pleiotropic effects, regulating the specification of cell fate, proliferation, self-renewal, survival, and apoptosis in a dose- and context-dependent fashion [1].

**Figure 1:**
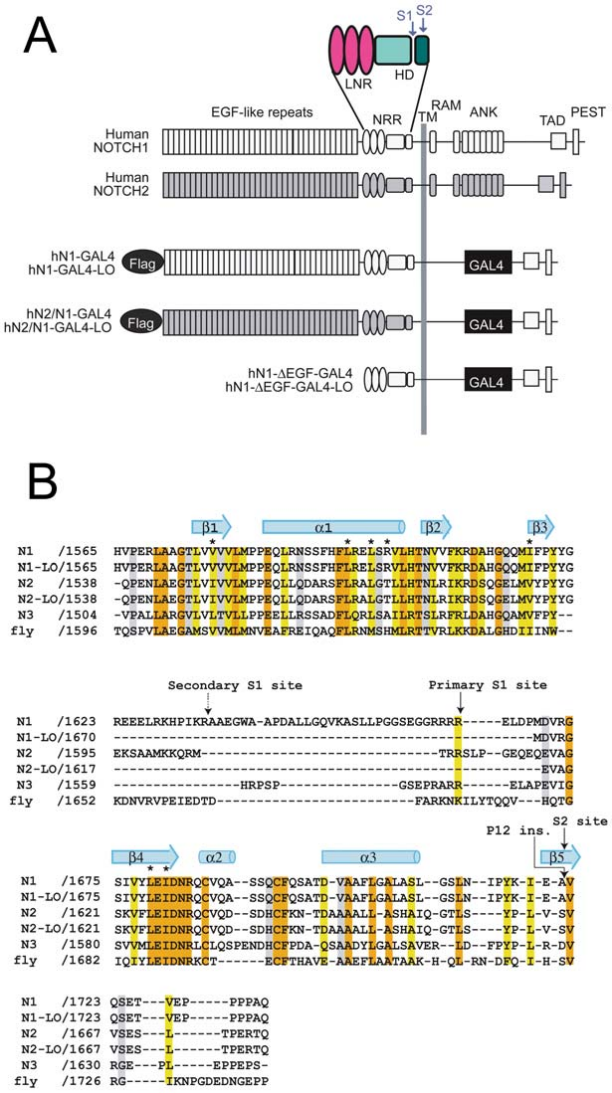
Notch domain organization, constructs used, and sequence alignment. A. Domain organization and construct design. Sequences derived from Notch1 are white, and sequences from Notch2 are gray. Chimeric Notch1 and Notch2 receptors were created by fusing the extracellular portion of either Notch1 (hN1-Gal4) or Notch2 (hN2/N1-GAL4) with the intracellular portion of Notch1 in which the RAM-ANK domain was replaced with the DNA binding domain of Gal4. Key: FLAG, N-terminal FLAG epitope tag; EGF-like repeats, epidermal growth factor-like repeats; NRR, negative regulatory region consisting of three Lin12/Notch repeats and the heterodimerization domain; TM, transmembrane; ANK, ankyrin repeats; TAD, transcriptional activation domain; PEST, degron domain rich in proline, glutamate, serine, and threonine residues; Gal4, DNA binding domain of the Gal4 transcription factor. B. Multiple sequence alignment of heterodimerization domain sequences of human Notch1–3, *Drosophila* Notch, and the N1 and N2 deletion constructs (N1-LO and N2-LO, respectively) used in these studies. The amino acid residues are colored according to the clustalW conservation convention: orange- absolute identity in all sequences, yellow- conserved substitution in one or more sequences, grey- semi-conserved substitution in one or more sequences. Alpha helix and beta sheet secondary structural elements of the heterodimerization domain are denoted with light blue cylinders or arrows, respectively. The S2 cleavage site and primary and secondary furin cleavage (S1) sites identified by *in vitro* furin cleavage of the human Notch1 NRR are marked with arrows. Tumor associated mutations analyzed are denoted by asterisks (point mutations) and an arrow (P12 insertion mutation).

Mammalian Notch receptors (Figure 1A) normally undergo proteolytic processing by a furin-like protease during maturation at a site termed S1 that lies about 70 amino acids external to the transmembrane segment [2], yielding two non-covalently associated extracellular (NEC) and transmembrane (NTM) subunits [2], [3], [4]. A series of 29–36 EGF-like repeats beginning at the N-terminal end of NEC constitute the ligand-binding domain of the receptor [5], [6], [7]. These EGF-like repeats are followed by three highly conserved LIN-12/Notch repeats (LNRs) and a subsequent “heterodimerization domain” (HD), which contains the S1 site as well as a second protease-cleavage site termed S2 [8]. The remainder of the NTM subunit consists of the transmembrane segment and the intracellular domain of Notch (ICN).

Together, the LNR and HD domains constitute the Notch negative regulatory region (NRR) [9], [10], [11], which maintains non-covalent association of the two subunits [8] and restrains the receptor in an autoinhibited, protease-resistant conformation prior to activation by ligands [12], [13]. Binding of ligands activates Notch by inducing sensitivity to metalloprotease cleavage at site S2, which lies about 12–13 amino acids external to the transmembrane domain [14], [15]. S2 cleavage is mediated by members of the ADAM family of metalloproteases, and is followed rapidly by cleavages within the transmembrane domain of NTM by γ-secretase [16], [17], [18]. Because the S2 site is deeply buried in the autoinhibited state [12], [13], binding of ligand to NEC must induce a conformational change in the NRR that exposes the S2 site and permits metalloprotease access.

The importance of the NRR in maintenance of the “off-state” is emphasized by the observation that mutations in this region cause aberrant Notch activation in species ranging from worms to man [11], [19]. In humans, roughly 40% of cases of T-cell acute lymphoblastic leukemia (T-ALL) harbor acquired mutations involving the NRR of Notch1 that take the form of point mutations and small in-frame insertions and deletions (see [20] for a recent review). These mutations can be classified based on whether they lie within the NRR core (class 1) or consist instead of insertions of 12 or more amino acids in the juxtamembrane regions immediately adjacent to the S2 site (class 2). The rare class 2 mutations are believed to create a deprotected S2 cleavage site, a model consistent with the observation that these mutations typically produce large, ligand-independent increases in Notch1 signaling activity. In contrast, the more common class 1 mutations are of lower, more variable strength and appear to destabilize the NRR [21]. Whether or not the activating effects of these mutations depend on S1 cleavage has not yet been examined.

The functional significance of cleavage by furin-like proteases varies among different transmembrane proteins; in some instances, cleavage is required for activity, whereas in others it has little or no effect. For example, class I viral envelope glycoproteins, which constitute the membrane fusion machinery of viruses like influenza and HIV, undergo an essential priming cleavage by a furin-like protease during maturation to generate the fusion-active protein. This cleavage step induces the transmembrane subunit to undergo a dramatic conformational change that allows its N-terminal end (also called the “fusion peptide”) to engage the target cell membrane in response to co-receptor (HIV) or low pH (influenza hemagglutinin) and initiate entry of the virus into the cell (see [22] for a recent review). In contrast, while low-density lipoprotein related protein (LRP-1) undergoes cleavage by furin during maturation and export to the cell surface [23], mutated forms that remain unprocessed bind, endocytose, and degrade ligands with kinetics indistinguishable from normal receptors, with the only reported effect of furin resistance apparently being slowed transport of LRP-1 to the cell surface [24].

Studies to date on the effect of S1 cleavage on Notch function have yielded disparate results and conclusions. Early studies of mammalian Notch receptors suggested that S1 cleavage is a prerequisite for delivery of receptors to the cell surface. Murine Notch1 receptors rendered resistant to furin cleavage by mutation of the primary S1-cleavage site on the carboxyl side of R1654 accumulate intracellularly in HeLa cells [2], and biotinylation studies failed to detect uncleaved precursor forms of Notch1 and Notch2 at the cell membrane [3]. Conversely, Weinmaster's group has shown that mutated Notch1 receptors resistant to cleavage at S1 are competent for cell surface expression [25], [26], but are apparently defective in terms of ligand-mediated activation of canonical Notch signaling, leading to the suggestion that dissociation of NTM may be a prerequisite for ligand-induced S2 cleavage. On the other hand, studies using furin-resistant receptors in the fly have suggested that S1 cleavage is not required for the function of *Drosophila* Notch [27]. Thus, no consensus has been reached about the role of S1 cleavage in Notch function. X-ray structures of the NRR regions from human Notch1 and Notch2, solved using polypeptides engineered to remove the S1 cleavage site, suggest that the S1 site of both receptors lies within a loop outside of the structural core of the HD domain [12], [13]. Moreover, both the length of the S1-cleavage loop and its amino acid sequence are poorly conserved in mammalian Notch receptors (Figure 1B), suggesting that the functional role of S1 cleavage in Notch signaling may not be straightforward, and might even vary according to the specific receptor being studied.

Here, we have determined the effects of S1 cleavage on the structure of the juxtamembrane portions of human Notch1 and Notch2, and have examined the effects of S1-resistance on the surface expression and ligand-mediated activation of these two receptors in quantitative assays. We find that S1 cleavage does not result in a substantial or large-scale change in the conformation of the Notch1 NRR, nor does it dramatically alter the conformation of the isolated Notch2 HD domain. For functional studies, S1 resistant receptors were engineered by deletion of the S1 cleavage loop using the structural studies as a guide (referred to as “loopout” or LO receptors; Figure 1B). While S1-resistant Notch1 receptors show increased intracellular retention, S1 cleavage is not absolutely required for surface expression or ligand-induced receptor activation. We also find that furin-resistance has little effect on ligand-independent Notch1 activation by a prototypic class 2 T-ALL-associated mutation, and only partially suppresses Notch1 activation by class 1 T-ALL-associated mutations. In contrast to Notch1 receptors, S1-resistant Notch2 receptors are fully competent for cell surface delivery and for activation by ligands. Together, these findings argue against a requisite role for S1 cleavage in physiologic or pathophysiologic Notch receptor activation, and highlight how subtle differences among Notch receptors in response to S1 cleavage may result in receptor-specific functional consequences.

## Results

### Structure of furin-cleaved human Notch1 NRR

To investigate the structural consequences of proteolysis of human Notch1 with furin, we determined the structure of the human Notch1 NRR by X-ray crystallography after *in vitro* furin cleavage. Mass spectrometric analysis of the furin-treated protein revealed that furin cleaves the loop in at least two places: after R1633 and after R1664. Crystals of the furin-cleaved protein grew in space group P4_3_2_1_2, and the structure of this protein was determined to 3.2 Å resolution (Table 1 and Supplemental Figure S1; pdb ID code 3I08).

**Table 1:** Data Collection, phasing, and refinement statistics.

Data collection	Human NOTCH1 NRR furin-cleaved
Space group	P4_3_2_1_2
Cell dimensions	
*a*, *b*, *c* (Å)	65.9, 65.9, 322.6
α,β,γ(°)	90, 90, 90
Resolution (Å)	50.0–3.2(3.26–3.20)*
*R*_sym_	13.2(51.8)
I/σI	9.9(2.3)
Completeness (%)	94.4(97.1)
Redundancy	5.4(5.6)
**Refinement**	
Resolution (Å)	46.3–3.2
No. reflections	11320
*R*_work_/*R*_free_	22.9/28.6
No. atoms	
Protein	3566
Ligand/ion	7
Water	21
*B*-factors (protein)	74.9
R.m.s deviations	
Bond lengths (Å)	0.015
Bond angles (°)	1.73

**Figure 2:**
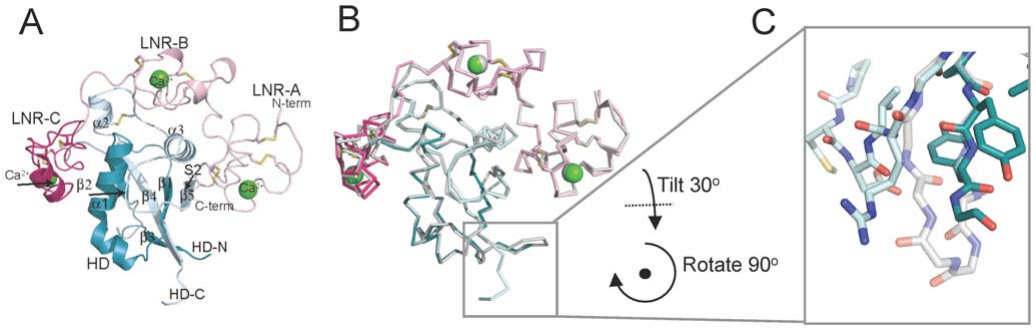
X-ray structure of the Notch1 NRR after furin-cleavage. A. Ribbon diagram of the structure of the furin-cleaved Notch1 NRR. The LNR modules are in shades of pink. The region of the HD domain that precedes the chain break at the S1 site is colored dark cyan, and the region after the cleavage site is in light blue. Calcium ions are shown as green spheres. The S2 site is indicated by an arrow. B. Overlay of the structure of the S1-cleaved Notch1 NRR (colors as in A) upon that of the Notch1 NRR lacking the S1-cleavage loop (gray; pdb ID code 3eto). C. Close-up view around the S1 site. The cleaved-Notch1 NRR is shown with colored sticks, and the backbone of the loop-deleted Notch1 NRR is shown in white for comparison.

The S1-cleaved NRR structure demonstrates that key interdomain interactions observed in the autoinhibited uncleaved Notch1 NRR (determined after deletion of the 47-residue loop containing the furin cleavage sites, [12]) are preserved in the S1-cleaved NRR. The major difference between the furin-cleaved protein and the protein harboring the loop deletion (hereafter N1-LO, for “loop out” deletion) lies in the immediate neighborhood of the S1 cleavage site (Figure 2), where the furin-treated protein has a chain break as a result of furin cleavage. Other minor variations between the two structures are present in the linker connecting LNR-B to LNR-C and in the surface exposed loops of LNR-A. These parts of the structure are modeled with higher B-factors than the rest of the model, suggesting that the observed differences result from intrinsic flexibility in these loops (Supplemental Figure S1B).

### NMR evaluation of normal and furin resistant forms of the human Notch2 HD domain

**Figure 3:**
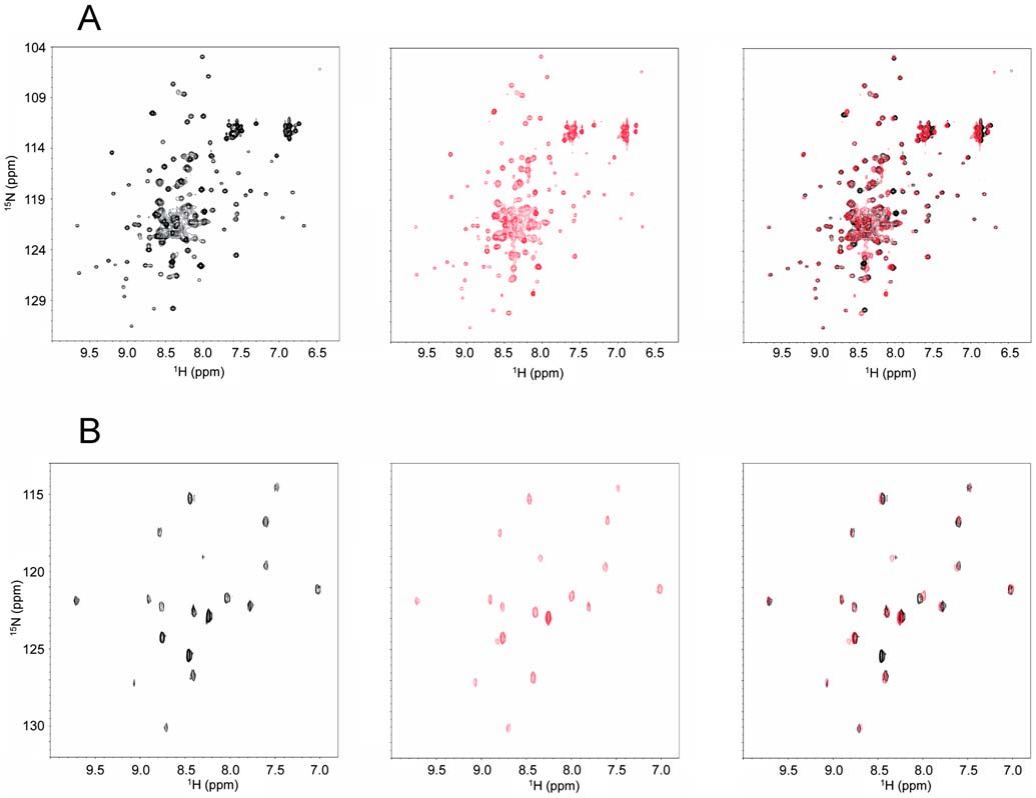
Furin cleavage does not affect the structure of the human Notch2 HD domain. (A) ^15^N HSQC spectrum of uniformly ^15^N-labeled human Notch2 HD domain before (black, left panel) and after (red, center panel) *in vitro* furin processing. A superposition of the two spectra is also shown (right panel). After acquisition of the initial spectrum of the human Notch2 HD domain (350 mM) at 18°C in 50 mM Bis-Tris (pH 7.0) containing 1 mM CaCl_2_, 50 mM NaCl, the protein was incubated for 13.5 hrs with 150 µl furin at 37°C. A second spectrum was then taken at 18°C. (B) HSQC spectrum of the human Notch2 HD domain selectively labeled with ^15^N-Leucine before (black, left panel) and after (red, center panel) furin cleavage. A superposition of the two spectra is also shown (right panel). Spectra in (B) were acquired at pH 6.0; all other conditions were identical to (A).

Because we were unable to grow diffracting crystals of the Notch2 NRR after furin cleavage, we monitored the conformational effects of cleaving Notch2 at the S1 site by solution NMR (Figure 3A). The ^15^N HSQC spectrum of the human Notch2 HD domain in its uncleaved form contains numerous overlapping peaks with random coil chemical shifts superimposed over a well-dispersed set of peaks. These features of the spectrum suggest that there are both disordered and well-structured regions in the HD domain precursor. Cleavage of the Notch2 HD domain by furin produced little detectable conformational change, as assessed by comparing the spectra acquired before (black) and after (red) cleavage. However, crowding and overlap in the middle of these spectra made it difficult to exclude the possibility that local conformational changes occur within the HD domain.

To eliminate crowding and overlap from the spectrum, we obtained HSQC spectra from precursor and furin-cleaved forms of the Notch2 HD domain after selective labeling with ^15^N-leucine. Because there are 19 leucine residues distributed fairly evenly throughout the HD sequence, they are well suited to report on local environmental alterations across the entire domain (Figure 3B). The spectra of the precursor and furin-treated Notch2 HD domain are virtually superimposable, with only one leucine exhibiting a chemical shift perturbation of more than 0.1 ppm in ^1^H and 0.5 ppm in ^15^N. The shifted residue, L1610, is located only two amino acids C-terminal to the S1 site and therefore expected to be affected by furin cleavage directly. Together, these results indicate that S1 cleavage has little effect on the overall conformation of the HD domain from human Notch2.

We next analyzed the furin sensitivity and NMR spectra of the human Notch2 HD domain after deletion of the S1-cleavage loop (hereafter referred to as N2-LO) to determine whether removal of the loop conferred *in vitro* resistance to furin cleavage or substantially affected the structure of the HD domain. The region of the Notch2 HD domain excised for these studies is the same 22-residue internal deletion (removing the S1 site and adjacent non-conserved residues) used to facilitate the crystallographic studies of the complete Notch2 NRR [13]. In contrast to the normal HD domain of human Notch2, which was efficiently cleaved into two species of the expected size, the loop-deleted form of the Notch2 HD domain was highly resistant to furin cleavage, even after overnight incubation at 30°C, based on analysis by SDS-PAGE (Supplemental Figure S2) and reversed-phase analytical HPLC.

**Figure 4:**
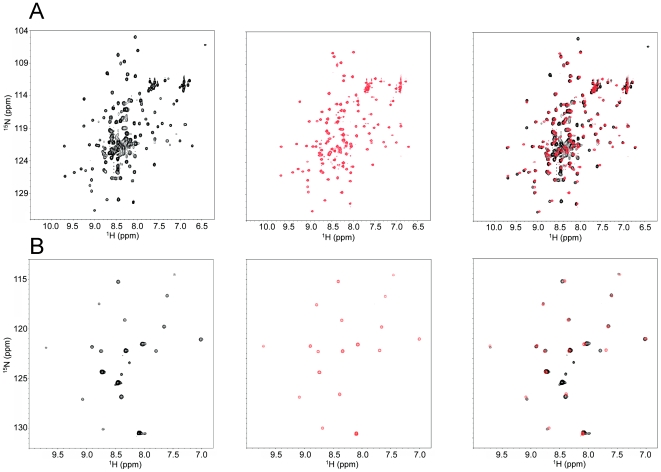
Removal of the S1 loop does not affect the structure of the human Notch2 (N2) HD domain. (A) Superposition of ^15^N HSQC spectra of wild-type (black, left panel) and N2-LO (red, center panel) HD domains. A superposition of the two spectra is also shown (right panel). Spectra were acquired at 20°C in 5 mM Bis-Tris (pH 7.0) containing 50 mM NaCl (wild-type) or 5 mM Bis-Tris (pH 6.6) containing 25 mM NaPO_4_, 50 mM NaCl (N2-LO). (B) Superposition of selectively ^15^N-Leucine labeled HSQC spectra of wild-type (black, left panel) and N2-LO (red, center panel) HD domains. A superposition of the two spectra is also shown (right panel). Spectra in (B) were acquired at 20°C in 5 mM Bis-Tris (pH 6.8) containing 50 mM NaCl.

To determine if the removal of the S1 cleavage loop perturbed the structure of the HD domain, we acquired an HSQC spectrum of a ^15^N-labeled sample of the N2-LO form of the HD domain and compared it with that of the normal human Notch2 HD domain (Figure 4A). Overall, the chemical shifts of the resonances in the N2-LO protein (Figure 4A, red) correspond closely to many of the dispersed peaks of the wild-type domain (Figure 4A, black), suggesting that N2-LO form of the HD domain contains much of the structured region present in the parent domain. Moreover, the significant decrease in the number of the degenerate peaks clustered in the random-coil region of the spectrum is consistent with the crystallographic studies of human Notch1, which suggest that the S1 cleavage site lies within a disordered region of the HD domain.

To cross-validate these findings, we also compared the spectrum of the N2-LO form of the Notch2 HD domain after selective ^15^N leucine labeling with the spectrum of the selectively labeled normal Notch2 HD domain. Again, the resonances of the N2-LO protein are nearly superimposable on most of the peaks from the wild-type protein (Figure 4B). One notable exception is the peak corresponding to L1610, which is present in the wild-type protein, but not in the N2-LO. These data reinforce the idea that the overall structure of the loop-deleted protein is highly similar to that of the wild-type HD domain.

### Construction and Evaluation of Isogenic Cell Lines Expressing Chimeric Notch Receptors

**Figure 5:**
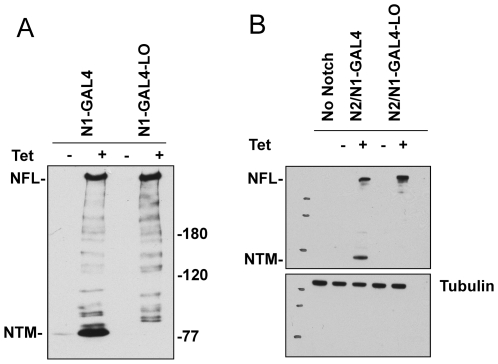
Western blots of normal and S1 cleavage-resistant Notch receptors. (A) Western blot of whole cell extracts of U2OS Flp-in stable cell lines showing tetracycline (Tet)-dependent expression of N1-GAL4 and the loop-deleted N1-GAL4 (hN1-GAL4-LO) chimeric receptors, and (B) Western blot of whole cell extracts of U2OS Flp-in stable cell lines showing tetracycline (Tet)-dependent expression of N2/N1-GAL4 and the loop-deleted N2/N1-GAL4 (N2/N1-GAL4-LO) chimeric receptors. Cells were incubated with Tet (1 mg/ml; +) or carrier alone (−) for 24 hr prior to lysis. Each blot was stained with a rabbit polyclonal antibody specific for the transcriptional activation domain of hN1 [34]. Key: NFL, full-length Notch; NTM, transmembrane subunit of the S1-cleaved receptor. In (B) the lower portion of the blot was stained for α-tubulin as a loading control.

Next, we investigated the effect of S1 resistance on surface expression and ligand-mediated signaling of Notch1 and Notch2. In order to do this as quantitatively as possible, we used isogenic cell lines that permit the tetracycline-inducible expression of proteins of interest from a single genomic FRT recombination site. We introduced into this site expression cassettes that encode chimeric Notch1 receptors consisting of (from N-terminus to C-terminus): the Notch1 leader peptide; a FLAG epitope tag; the ectodomains and transmembrane domain of Notch1 or Notch2, with or without the loop-deletion mutations identical to those used in structural studies that confer S1-resistance; and a common intracellular domain consisting of the DNA-binding domain of Gal4, the strong transcriptional activation domain of Notch1, and the C-terminal PEST domain of Notch1 (Figure 1A). These proteins are referred to as N1-GAL4, N1-GAL4-LO, N2/N1-GAL4, and N2/N1-GAL4-LO, respectively. The rationale for using these chimeric receptors was twofold. First, the Gal4 reporter genes have negligible intrinsic activity in mammalian cells, thus providing excellent signal to noise ratios in ligand-dependent activation assays while avoiding the confounding influence of signals generated by endogenous Notch receptors [28]. Second, the use of chimeras with identical intracellular regions ensures that differences in surface delivery or signaling activity can be directly attributed to differences in the Notch1 and Notch2 ectodomains. Western blot analysis of these four cell lines confirmed that chimeric Notch receptor expression was dependent on tetracycline, and that S1 cleavage of the Notch1 and Notch2 loop-deletion proteins was completely undetectable (Figure 5A,B).

### Effect of Loop Deletion on the Trafficking of Notch1 and Notch2

**Figure 6:**
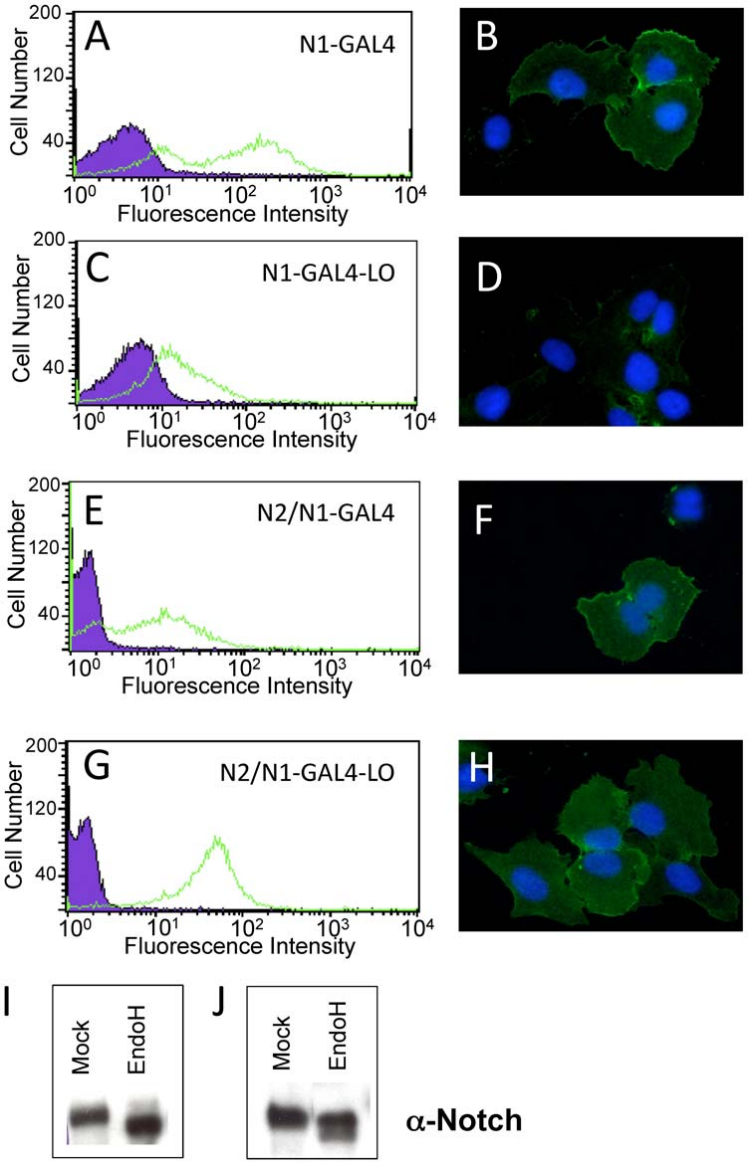
S1-cleavage-resistant Notch receptors are expressed on the cell surface. Surface receptors were stained with a FITC-conjugated anti-FLAG antibody and detected either by flow cytometry or fluorescence microscopy. (A, B). Surface levels of the N1-Gal4 chimeric receptor, detected by flow cytometry (A) and fluorescence microscopy (B). (C, D). Surface levels of the N1-Gal4-LO chimeric receptor, detected by flow cytometry (C) and fluorescence microscopy (D). (E, F). Surface levels of the N2/N1-Gal4 chimeric receptor, detected by flow cytometry (E) and fluorescence microscopy (F). (G, H). Surface levels of the N2/N1-Gal4-LO chimeric receptor, detected by flow cytometry (G) and fluorescence microscopy (H). In all flow cytometry plots, the green trace corresponds to staining with the FITC-conjugated anti-FLAG antibody, and the black and purple plot shows staining with an isotype-matched control antibody. In the immunofluorescence panels, the surface Notch receptors are stained with anti-FLAG (green), and the nuclei (blue) are stained with DAPI. (I, J) Western blot analysis of Endo H sensitivity. Whole cell lysates from stably transfected U2OS cells expressing the N1-GAL4-LO protein (I) or the N2/N1-GAL4-LO protein (J) were either mock treated (left) or incubated with Endo H glycosidase before analysis (right).

We next compared the surface expression of the various Notch receptors by flow cytometry and immunofluorescence microscopy using antibodies against the extracellular FLAG epitope tag (Fig. 6A–H). The S1-competent Notch1-Gal4 chimera is delivered readily to the cell surface upon tetracycline induction (Fig. 6A,B). In contrast, steady state cell-surface levels of the S1-resistant, N1-GAL4-LO protein are 5–10-fold lower after induction (Fig. 6C,D), despite comparable levels of overall expression as judged by Western blotting (Fig. 5B). Examination of the overall level of expression by flow cytometry using the same antibody after cell permeabilization indicates that the total protein levels are comparable in these two cell lines (Supplemental Figure S3). Additional biochemical analyses showed that a high fraction of the total N1-GAL4-LO protein was sensitive to the glycosidase EndoH, which is consistent with retention of these polypeptides in the ER/Golgi compartment (Fig. 6I). Immunofluorescent staining of permeabilized cells also showed increased co-localization of the N1-GAL4-LO mutant and calreticulin, an ER marker, when compared to cells expressing N1-GAL4 (Supplemental Figure S4). Thus, the reduced surface levels of the S1 cleavage-resistant form of Notch1 results from a defect in the transport of the proteins to the cell surface, an observation consistent with previous studies (*e.g*. [2]).

In contrast to Notch1, N2/N1-GAL4 and the S1-resistant N2/N1-GAL4-LO protein are both readily detected at the cell surface by both flow cytometry and immunofluorescence (Fig. 6E–H). Flow cytometry analysis indicated that the levels of the S1-resistant Notch2 protein are somewhat higher than the surface levels of N2/N1-GAL4 (compare Figure 6E with 6G ), even though the overall expression levels are not substantially different between the two cell lines based on Western blotting (Figure 5C) and flow cytometry studies after cell permeabilization (Supplemental Figure S5). Most of the S1-resistant Notch2 molecules were also resistant to cleavage with EndoH (Figure 6J), consistent with the localization studies.

### Effect of Loop Deletion on the Ligand-Mediated Activation of Notch1 and Notch2

**Figure 7:**
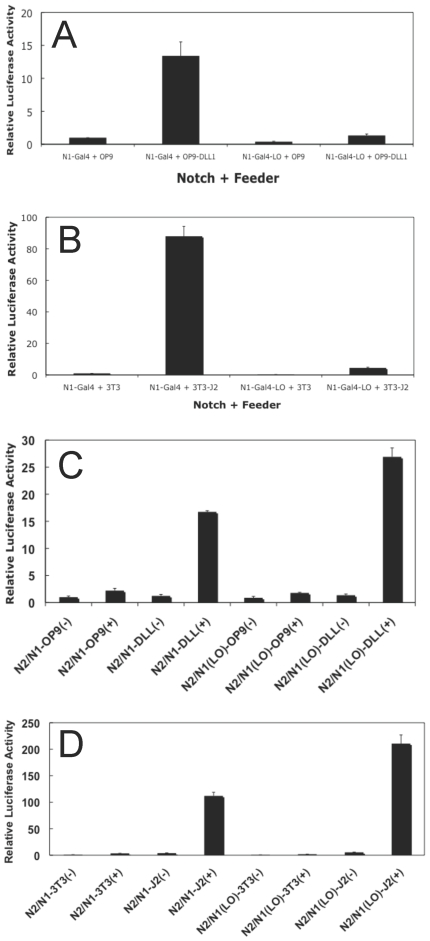
Activation of S1-cleavage resistant Notch receptors by ligands of the Delta and Jagged families. (A) Isogenic U2OS flp-in cell lines that express N1-Gal4 or N1-Gal4-LO receptors under the control of tetracycline were transfected with a Gal4 responsive luciferase reporter plasmid and co-cultured with OP9 or OP9-Delta-like-1 feeder cells in the presence of tetracycline for 24 hr. Dual luciferase activities were measured in whole cell lysates. In (B), the experiment is identical to that in (A), except that 3T3 or 3T3-Jagged2 feeder cells were used. (C) Isogenic U2OS flp-in cell lines that express N2/N1-Gal4 or N2/N1-Gal4-LO receptors under the control of tetracycline were transfected with a Gal4 responsive luciferase reporter plasmid and co-cultured with OP9 or OP9-Delta-like-1 feeder cells in the presence (+) or absence (−) of tetracycline for 24 hr. Dual luciferase activities were measured in whole cell lysates. In (D), the experiment is identical to that in (C), except that 3T3 or 3T3-Jagged2 feeder cells were used.

In the case of Notch1, a decreased response of the loop-deletion mutant to both Jagged-2 and Delta-like-1 ligands is observed in co-culture reporter gene activation assays, as predicted by the reduced number of receptor molecules at the cell surface (Figure 7A,B). However, γ-secretase dependent, ligand-mediated activation of N1-GAL4-LO receptors was still readily detectable, indicating that some of the receptors reaching the cell surface are competent for activation (Supplemental Figure S6).

The S1-resistant, N2/N1-GAL4-LO chimeras exhibited strong activation of a Gal4-luciferase reporter gene in co-culture assays with cells expressing either Delta-like-1 or Jagged2 (Figure 7C,D and Supplemental Figure S6). In fact, the level of activation was generally somewhat higher in the cells expressing the chimeric Notch2 loop-deleted receptors than in the cells expressing the chimeric N2/N1-GAL4 receptors with normal Notch2 ectodomains, as predicted based on the flow cytometry studies showing that more of the Notch2 molecules with the loop deletion are seen at the cell surface. Taken together with the trafficking studies, these findings indicate that the susceptibility of uncleaved Notch1 to intracellular retention is receptor-specific, and that the attenuated signaling seen in S1-cleavage-resistant Notch1 receptors is not a general property of Notch receptors.

### Effect of S1-Resistance on NRR Mutation-Mediated Activation of Notch1

**Figure 8:**
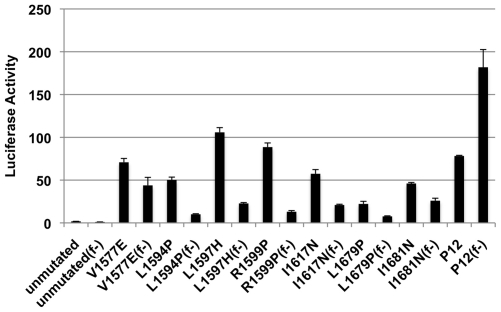
Effect of S1-resistance on ligand-independent activation associated with T-ALL mutations of human Notch1. U2OS cells were transiently transfected with ΔEGF receptors containing T-ALL associated mutations alone or in *cis* with the S1-resistant loop-deletion mutation (f-; for furin-resistant). All data points were obtained in triplicate, with error bars indicating the standard deviation of the three replicates.

We also investigated the effect of S1-resistance on Notch1 activation by a panel of T-ALL associated NRR mutations of various classes (Figure 8). These mutations were introduced into Notch1 receptors lacking the ligand-binding EGF repeat region, so as to permit measurement of ligand-independent receptor activation, and compared for signaling strength in transiently transfected U2OS cells. Furin resistance partially suppressed Notch1 activation by class 1 mutations, yet all mutations tested retained some ligand-independent signaling activity in the absence of furin cleavage. In contrast, furin resistance was reproducibly associated with increased activation of Notch1 receptors bearing a class 2 mutation originally identified in the cell line P12-Ichikawa, which contains a 14-residue insertion immediately preceding the transmembrane segment. We conclude that there is no obligate role for furin cleavage in the activation of Notch1 by T-ALL-related NRR mutations, indicating that, as with ligand-mediated Notch activation, these mutations can activate signaling by causing conformational changes in the NRR, regardless of whether or not subunit dissociation can take place.

## Discussion

The work reported here examines the effect of S1 cleavage on the structure and activity of Notch1 and Notch2. The structural studies clarify how S1 cleavage affects the conformation of the Notch1 NRR, and the studies of trafficking and signaling by Notch1 and Notch2 receptors engineered to be S1 resistant highlight the difficulties in generalizing conclusions based on studies of single Notch receptor family members.

Previously reported X-ray structures of the Notch1 and Notch2 negative regulatory regions were determined using recombinant proteins with deletions that removed the S1-cleavage loop. In both structures, the S1 cleavage site was predicted to lie in a loop connecting beta-strands 3 and 4, more than 17 Å away from the S2 site and well removed from all other interdomain contacts.

The X-ray structure of the furin-cleaved Notch1 NRR clearly shows that S1 cleavage only causes minor local perturbations in the conformation of the NRR, which remains in its autoinhibited form after processing at S1 (Figure 2). Biophysical characterization of the isolated Notch2 HD domain by NMR also confirms that the structure of the HD domain is minimally perturbed by furin cleavage or by excision of the S1 loop. The NMR studies suggest that the S1 cleavage site in Notch2 also lies within an unstructured region of the HD domain and show that furin cleavage does not contribute to the proper folding of the isolated HD domain (Figures 3 and 4). Although these results may have been predicted based on inspection of the loop-deleted receptors, these studies provide the first direct evidence that S1 cleavage exerts little effect on the conformation of either Notch1 or Notch2.

A more surprising finding is that there is a differential effect on signaling and trafficking when S1 resistance is engineered into the Notch1 and Notch2 receptors. Notch2 receptors lacking the S1 loop appear to traffic normally to the cell surface and are activated by ligand to a comparable or greater degree than normal receptors. On the other hand, Notch1 receptors lacking the S1 loop exhibit a substantial defect in transport to the cell surface, with steady state cell-surface levels reduced by 5–10-fold, even though there are comparable levels of overall expression. Despite the reduced number of S1-resistant Notch1 receptors at the surface, uncleaved Notch1 receptors retain their ability to signal, either in response to ligand or when combined in *cis* with a T-ALL associated mutation. The persistent strong activity of the class 2 mutation tested here in the context of the loop deletion suggests the possibility that activating proteolysis of these cancer-associated mutations occurs prior to surface delivery, somewhere in the late ER or Golgi. The hypomorphic activity of an S1-resistant receptor that closely resembles the Notch1 variant investigated here is also consistent with the reduced surface expression and signaling activity we observe with our S1-resistant protein [26]. Overall, despite the qualitative differences between Notch1 and Notch2, our data, in combination with published studies [26], [27] support the conclusion that S1 cleavage is not absolutely required for conveying Notch signals. Though unlikely, it is even possible that differential susceptibility of uncleaved N1 and N2 receptors to intracellular retention allows the expression level of furin-like proteases to influence N1 vs. N2 signaling in cells where both proteins are being expressed.

Studies by Artavanis-Tsakonas and colleagues in an accompanying manuscript also point to complexity in analyzing the role of S1 cleavage in *Drosophila* Notch. The *Drosophila* protein has two potential sites for S1 cleavage, based on analysis of the primary amino-acid sequence. Structure-based sequence alignment places the first potential site, RLKK (*Drosophila* residue numbering 1637–1640; Figure 1B), at a location analogous to the end of beta-strand two and in the connecting segment to strand three (Figure 2), and the second potential site, RKNK (*Drosophila* residue numbering 1667–1670) in the unstructured loop that aligns to the region that contains the mammalian S1 cleavage site. Neither mutation of the RKNK sequence or loop deletion, however, prevents cleavage of the fly protein into extracellular and transmembrane subunits. Though mutation of the RLKK site in the fly protein creates a receptor that is not processed, active, or observed at the cell surface, it is not certain whether this outcome is a secondary consequence of global structural disruption, or a result of a critical dependence on S1 cleavage for Notch function in *Drosophila*.

The lack of an absolute requirement for S1 cleavage in promoting the maturation of mammalian Notch receptors is consistent with a number of prior studies, and is easily reconciled with others in light of recent structural and biochemical advances. In chimeric mouse/fly receptors expressed in 293T cells, replacement of the mouse Notch1 S1-cleavage loop by the *Drosophila* loop region results in expression of the full-length protein, rather than the S1 cleaved form, on the cell surface [27]. Additional studies detecting low levels of uncleaved Notch at the cell surface [29], [30] reinforce our conclusion that S1 cleavage is normally a marker of, rather than a prerequisite for, mammalian Notch receptor maturation and cell-surface delivery.

Older mutational studies suggesting that S1 cleavage may be required for maturation of mammalian Notch receptors and for canonical signaling may have been confounded by inadvertent disruption of the structural integrity of the HD domain. In two of these studies [25], [27], S1 cleavage was abolished by deleting not only the cleavage sites, but also by eliminating the most highly conserved sequence in the HD domain, the LEIDNR sequence, which in human Notch2 lies on a beta-strand from residues 1624–1630 right in the core of the HD domain [13]. Thus, it is virtually certain that these mutations had additional unintended effects beyond simple inhibition of cleavage at site S1.

What then is the role of cleavage of mammalian Notch receptors by furin-like proteases? It seems likely that S1 cleavage of receptors undergoing transport through the Golgi has some specific functional importance, because the cleavage site is present in mammalian Notches 1–3. One additional possibility is that S1 cleavage facilitates proper down-regulation of Notch receptors. The extracellular part of Notch1 and Notch2 (like other Notch receptors) is very large and includes thirty-nine disulfide-bonded domains (36 EGF-like repeats and 3 LNRs) that are expected to require reduction before becoming sensitive to proteolytic degradation during turnover. Retro-translocation and endoplasmic reticulum associated degradation, or lysosomal degradation, would pose a formidable challenge if there were no S1 cleavage event to decouple the extracellular and transmembrane subunits. Perhaps S1 cleavage thus allows for efficient disposal of the NTM subunit by dissociating it from the rest of the extracellular part of the receptor. In this case, a cleavage site (S1) that is distinct from activating cleavage sites (S2 and S3) would allow cellular degradation machinery to distinguish receptors designated for destruction from those designated for proteolytic activation. Further studies are needed to test this possibility and to understand the biological role of S1 cleavage in Notch receptor function.

## Materials and Methods

### Recombinant protein expression and purification

A plasmid encoding the human Notch1 NRR (residues E1446-Q1733; Genbank ID 148833507) was modified to contain a N-terminal hexahistidine tag followed by a TEV cleavage site. A plasmid encoding the human Notch2 heterodimerization domain (Notch2 HD domain, corresponding to residues E1540 to L1678, Genbank ID 24041035) was constructed by subcloning the corresponding PCR amplified sequence from the human Notch2 cDNA into a derivative of the pET21a(+) bacterial expression vector (Novagen) modified to contain an N-terminal hexahistadine tag followed by a TEV cleavage site.

The Notch1 NRR precursor was prepared essentially as described previously for the loop-deleted form of the Notch1 NRR [12]. The Notch1 NRR (1 mg/ml) was then cleaved *in vitro* overnight at 22°C in a total volume of 500 mL using 40 Units of recombinant furin (New England Biolabs, Beverly MA) in 10 mM Tris buffer, pH 8, containing 10 mM NaCl, and 1 mM CaCl_2_. The extent of cleavage was typically assessed by either SDS-page or reversed-phase HPLC. When the precursor was no longer detectable on SDS-PAGE, the furin-cleaved protein was then purified by size exclusion chromatography in 25 mM Tris buffer, pH 8, containing 100 mM NaCl and 10 mM CaCl_2_.

The S1 cleavage loop (residues K1596-E1617) was deleted from the Notch2 HD by directed mutagenesis (Stratagene). All encoded proteins were expressed in *E. coli* host strains (Novagen) BL21(DE3) or BL21(DE3)pLysS. Cell cultures were typically grown to 0.6 (A600) in Luria-Bertani broth at 37°C before the addition of 0.4 mM isopropyl-1-thio-β-D-galactopyranoside to induce expression of unlabeled Notch1 or Notch2 proteins. Uniformly ^15^N labeled proteins were expressed from cells grown in M9T minimal medium with ^15^NH_4_Cl as the sole source of nitrogen. Specific labeling with ^15^N-Leu was accomplished by inoculating a modified M9 minimal medium that contained natural abundance ammonium chloride supplemented with 200 mg/L of each unlabeled amino acid except leucine and 150 mg/L^ 15^N-Leu [31]. After induction for 4 h, cells were recovered by centrifugation, and resuspended in ice-cold 50 mM Tris buffer (pH 8.0) containing 300 mM NaCl, 20% sucrose (w/v), 10 mM β-mercaptoethanol, 0.5 mM EDTA, 10 mM imidazole, 0.2 mM PMSF, 2 mg/mL pepstatin, 5 mg/mL aprotonin, and 2 mg/mL leupeptin. Cells were lysed by freezing and thawing, followed by three cycles of sonication for 30 sec on ice. Insoluble material was removed from the lysate by centrifugation and the supernatant was adsorbed to nickel-nitrilotriacetic acid (Ni-NTA) agarose resin (Qiagen) at 4°C for 2 h. After extensive washing with 50 mM Tris buffer (pH 8.0) containing 300 mM NaCl and 10 mM imidazole, the bound histidine-tagged polypeptides were eluted from the Ni-NTA agarose resin with 50 mM Tris (pH 8.0) 300 mM NaCl, and 250 mM imidazole, After dialysis at 4°C for 12–16 h against 50 mM Tris (pH 8.0) containing 300 mM NaCl, human Notch2 HD constructs were further purified by size exclusion chromatography on a Superdex 75 column (Amersham Biosciences).

### X-ray crystallography of the furin-cleaved Notch1 NRR

After size exclusion, the S1-cleaved Notch1 NRR was concentrated to 10 mg/ml and subjected to crystallization trials. The protein crystallized in a buffer of 100 mM Sodium acetate, pH 4.0, containing 2 M NaCl and 10% glycerol. The crystals were cryoprotected in a buffer of 100 mM sodium acetate, pH 4.0, containing 2.5 M NaCl, and 35% glycerol, and the native dataset used to determine the structure was collected at APS beamline ID-24E. Data were reduced using HKL2000.

The furin-cleaved Notch1 NRR crystallized in the P4_3_2_1_2 space group with 2 molecules per asymmetric unit. The structure was solved by employing the structure of the loop-deleted Notch1 NRR as a search model in the program Phaser. F_o_-F_c_ and composite omit maps confirmed the presence of additional electron density in the region of the loop that was not present in the loop-deleted protein used as the initial model. Model building was performed using a combination of CNS and Refmac using NCS restraints. Data collection and refinement statistics are reported in Table 1. The coordinates were deposited in the protein data bank with ID code 3I08.

### Nuclear Magnetic Resonance Spectroscopy

Structural assessment of soluble human Notch2 HD domains was carried out using ^15^N HSQC experiments. ^15^N HSQC spectra of the human Notch2 HD domain before and after furin cleavage were acquired on a Varian UnityInova 600 spectrometer equipped with a cryogenic probe. All spectra were acquired at 18°C on 300 mL of ∼350 mM ^15^N-labeled protein samples in 90% H_2_O / 10% D_2_O containing 50 mM Bis-Tris buffer (pH 6) and 1 mM CaCl_2_, using a triple-resonance cryoprobe and an ^15^N HSQC pulse sequence with gradient water suppression. Data were processed with NMRPipe [32] and analyzed using NMRView [33]. ^1^H chemical shifts were directly calibrated using ∼0.3 mM 2,2-dimethyl-2-silapentane-5-sulfonate (DSS) as an internal standard. ^15^N chemical shifts were referenced indirectly to DSS. Furin cleavage was carried out on the same ^15^N labeled sample that was used to acquire the precleavage spectrum, by addition of furin directly into the NMR tube and monitoring cleavage through a set of HSQC experiments at 37°C. The final cleaved spectrum was acquired under the same conditions as the precursor sample and complete cleavage of the sample was verified by reversed-phase high performance liquid chromatography.

### Notch Mammalian Expression Plasmids

Modified cDNAs encoding various Notch receptors were assembled from full-length Notch cDNAs using PCR fragments and naturally occurring or engineered restriction sites. All portions of cDNAs created with PCR were confirmed by DNA sequencing. To permit detection of full-length Notch1 or Notch2 receptors on the cell surface, a single FLAG-epitope tag was inserted between the signal peptide and the first EGF-like repeat. For Notch2, the amino acid sequence of the resulting “joint” between these regions is AAAGDYKDDDDKGHALQC, where A is the C-terminal end of the human Notch2 signal peptide, and C is the first residue in EGF-like repeat 1 of human Notch2. cDNAs encoding tagged human Notch2 ectodomains (with and without the S1-cleavage-resistant “loopout” deletion) were then ligated through a unique Bsu36I site to cDNAs encoding a Gal4-ICN1 fusion cDNA, in which the coding regions for the RAM and ANK domains of ICN1 have been replaced by the Gal4 DNA binding domain. These molecules retain the strong Notch1 transcriptional activation domain, which can be detected on Western blots with a well-characterized rabbit polyclonal antibody [34]. To generate stable cell lines, the N1-Gal4, N1-Gal4-LO, N2/N1-Gal4, and N2/N1-Gal4-LO cDNAs were subcloned into the vector pcDNA5-FRT-TO (Invitrogen) and transfected into U2OS TRex cells (a kind gift of Jeff Parvin, Ohio State University) together with the plasmid pOG44 (Invitrogen), which encodes Flp recombinase. This strain of U2OS cells expresses the Tet repressor and contains a single genomic FRT site, which permits creation of isogenic recombinants containing a single transgene under the control of tetracycline. Stably transfected cells were selected with hygromycin.

### Western Blotting, Immunofluoresence Microscopy, and Flow Cytometry

Western blotting for Notch polypeptides was performed as described [21]. For immunofluorescent localization studies, tetracycline inducible U2OS Flp-In cell lines engineered to express Notch1 and Notch2 chimeric polypeptides were plated onto 25 mm coverslips in 6 well plates and incubated in presence of tetracycline (1 µM) in a 6 well plate at 37°C for 16 hr. Cells were fixed with 3.7% paraformaldehyde in phosphate-buffered saline (PBS) for 5 minutes at room temperature, and either stained directly or permeabilized for 5 minutes with PBS-0.2% Triton X-100. Coverslips were blocked for 30 minutes with PBS containing 4% non-immune rabbit serum. To detect FLAG-tagged Notch polypeptides, coverslips were incubated with anti-FLAG (M2 clone, Sigma, 1∶250) for 20 minutes at room temperature, washed three times for 5 minutes with PBS, incubated with rabbit anti-mouse antibody conjugated to FITC (Dako, 1∶250) for 20 minutes at room temperature, and washed again as before. To detect the endoplasmic reticulum polypeptide calreticulin, coverslips were incubated with rabbit anti-calreticulin (Abcam, 1∶250) for 20 minutes at room temperature, washed with PBS, and then incubated with donkey anti-rabbit antibody conjugated to rhodamine (Jackson Labs, 1∶5000) for an additional 20 minutes. Stained coverslips were mounted onto microscope slides with 15 µL of Prolong Fade Gold Reagent (Invitrogen), and then imaged and photographed on a Nikon Eclipse 2000-S fluorescence microscope linked to a Spot digital camera. For flow cytometry, U2OS flp-in cells expressing various FLAG-tagged Notch polypeptides were trypsinized to create single cell suspensions. To detect Notch polypeptides on the cell surface, cells were blocked in PBS containing 4% fetal bovine serum for 15 minutes on ice and then incubated with mouse anti-FLAG conjugated to Sure-Light APC (1∶160) or APC-conjugated isotype-matched murine control IgG (Perkin-Elmer) for 20 minutes on ice. Cells were then briefly counterstained with propridium iodide to exclude dead cells from the analysis. To detect intracellular Notch polypeptides, cells were treated according to instructions accompanying the Becton-Dickenson Permeabilization and Fixation kit. Permeabilized cells were blocked by incubation in PBS containing 10% goat serum for 15 minutes on ice and then incubated with anti-FLAG or control APC-conjugated antibodies as above. Data were acquired on a Becton-Dickenson FACSCalibur flow cytometer and analyzed using CellQuest software.

### Endoglycosidase Treatment of Notch Receptors

Cell lysates of N1-GAL4-LO and N2/N1-GAL4-LO were prepared for glycosidase treatment by lysing cells from a 6-well dish *in situ* with buffer containing 1% NP-40. The mock and EndoH samples were first denatured by boiling for 5 minutes in denaturation buffer (NEB). Appropriate reaction buffers and EndoH (NEB) were then added and the mock and EndoH sample incubated at 37°C for 2 hours. SDS loading buffer was added and samples run on a 6% Tris-glycine gel.

### Ligand Stimulation Assays

U2OS Flp-in N1-Gal4, N1-GAL4-LO, N2/N1-Gal4, and N2/N1-GAL4-LO cells were tested for ligand-dependent responsiveness as follows. On day 1, cells in 60 mm dishes of U2OS Flp-in cells were transfected with a mixture containing 1 mg of a Gal4-firefly luciferase reporter and 20 ng of an internal control pRL-TK Renilla luciferase plasmid (Invitrogen). On day 2, the U2OS Flp-in cells were split onto either OP9 or OP9-DLL1 cells (a kind gift of Dr. Juan-Carlos Zuniga-Pflücker) in the presence of absence of 1 mg/ml of tetracycline or carrier (70% ethanol) alone. On day 3 following 24 h of co-culture, firefly and *Renilla* luciferase activities were measured in whole cell extracts using the Dual Luciferase kit (Promega) and a specially configured luminometer (Turner Systems). The same procedure was used to examine responsiveness to the ligand Jagged2, comparing the reporter gene response of the Notch-expressing cells to 3T3 cells alone with that of 3T3 cells stably expressing Jagged2. In some experiments, the cells were treated post-transfection with the γ-secretase inhibitor compound E (a kind gift of Dr. Michael Wolfe) at 1 µM, or with carrier alone (0.01% DMSO). All data points within experiments were obtained in triplicate, and all experiments were repeated three times.

## Supporting Information

Figure S1 Electron density and ribbon diagram colored by B-factor. A. 2F_o_-F_c_ electron density map of the furin loop region, contoured to a level of 1.2 σ. B. Ribbon diagram of the furin-cleaved N1-NRR colored by B-factor in Pymol in a continuum of colors from low B- factors (blue) to high B-factors (red).
(0.95 MB PDF)

Figure S2 *In vitro* processing of the hN2 HD domain requires the S1 loop. (A) Normal human Notch2 HD (lanes 2 and 3) and human Notch2 HD-loopout (lanes 4 and 5) constructs before (lanes 2 and 4) and after (lanes 3 and 5) overnight cleavage at 37°C with recombinant furin. Lane 1 consists of molecular weight standards.
(0.15 MB PDF)

Figure S3 A-D. Flow cytometry of the indicated Notch-expressing cell lines after permeabilization. Receptors were detected with a FITC-conjugated anti-FLAG antibody (green). An isotype-matched antibody was used as a control (black and purple plots).
(0.47 MB TIF)

Figure S4 Immunofluorescent staining of U2OS cells stably transfected with Notch1-GAL4 (top) and Notch1-GAL4-LO (bottom) receptors.
(1.67 MB TIF)

Figure S5 Immunofluorescent staining of U2OS cells stably transfected with Notch2/N1-GAL4 (top) and Notch2/N1-GAL4-LO (bottom) receptors.
(1.62 MB TIF)

Figure S6 Activation of S1-cleavage-resistant Notch receptors is prevented by γ-secretase inhibitors. A. Chimeric Notch1 receptor activation was assessed in the presence (+) or absence (-) of the γ-secretase inhibitor compound E (1 µM). B. Chimeric Notch2 receptor activation was assessed in the presence (+) or absence (−) of the γ -secretase inhibitor compound E (1 µM). All data points were obtained in triplicate, with error bars indicating the standard deviation of the three replicates. Representative results from at least three independent experiments are shown.
(0.22 MB TIF)

## References

[1] Bray SJ (2006). Notch signalling: a simple pathway becomes complex.. Nat Rev Mol Cell Biol.

[2] Logeat F, Bessia C, Brou C, LeBail O, Jarriault S (1998). The Notch1 receptor is cleaved constitutively by a furin-like convertase.. Proc Natl Acad Sci U S A.

[3] Blaumueller CM, Qi H, Zagouras P, Artavanis-Tsakonas S (1997). Intracellular cleavage of Notch leads to a heterodimeric receptor on the plasma membrane.. Cell.

[4] Rand MD, Grimm LM, Artavanis-Tsakonas S, Patriub V, Blacklow SC (2000). Calcium depletion dissociates and activates heterodimeric notch receptors.. Mol Cell Biol.

[5] Rebay I, Fleming RJ, Fehon RG, Cherbas L, Cherbas P (1991). Specific EGF repeats of Notch mediate interactions with Delta and Serrate: implications for Notch as a multifunctional receptor.. Cell.

[6] Lawrence N, Klein T, Brennan K, Martinez Arias A (2000). Structural requirements for notch signalling with delta and serrate during the development and patterning of the wing disc of Drosophila.. Development.

[7] Xu A, Lei L, Irvine KD (2005). Regions of Drosophila Notch that contribute to ligand binding and the modulatory influence of Fringe.. J Biol Chem.

[8] Sanchez-Irizarry C, Carpenter AC, Weng AP, Pear WS, Aster JC (2004). Notch subunit heterodimerization and prevention of ligand-independent proteolytic activation depend, respectively, on a novel domain and the LNR repeats.. Mol Cell Biol.

[9] Kopan R, Schroeter EH, Weintraub H, Nye JS (1996). Signal transduction by activated mNotch: importance of proteolytic processing and its regulation by the extracellular domain.. Proc Natl Acad Sci U S A.

[10] Lieber T, Kidd S, Alcamo E, Corbin V, Young MW (1993). Antineurogenic phenotypes induced by truncated Notch proteins indicate a role in signal transduction and may point to a novel function for Notch in nuclei.. Genes Dev.

[11] Greenwald I, Seydoux G (1990). Analysis of gain-of-function mutations of the lin-12 gene of Caenorhabditis elegans.. Nature.

[12] Gordon WR, Roy M, Vardar-Ulu D, Garfinkel M, Mansour MR (2009). Structure of the Notch1-negative regulatory region: implications for normal activation and pathogenic signaling in T-ALL.. Blood.

[13] Gordon WR, Vardar-Ulu D, Histen G, Sanchez-Irizarry C, Aster JC (2007). Structural basis for autoinhibition of Notch.. Nat Struct Mol Biol.

[14] Mumm JS, Schroeter EH, Saxena MT, Griesemer A, Tian X (2000). A ligand-induced extracellular cleavage regulates gamma-secretase-like proteolytic activation of Notch1.. Mol Cell.

[15] Brou C, Logeat F, Gupta N, Bessia C, LeBail O (2000). A novel proteolytic cleavage involved in Notch signaling: the role of the disintegrin-metalloprotease TACE.. Mol Cell.

[16] De Strooper B, Annaert W, Cupers P, Saftig P, Craessaerts K (1999). A presenilin-1-dependent gamma-secretase-like protease mediates release of Notch intracellular domain.. Nature.

[17] Struhl G, Greenwald I (1999). Presenilin is required for activity and nuclear access of Notch in Drosophila.. Nature.

[18] Ye Y, Lukinova N, Fortini ME (1999). Neurogenic phenotypes and altered Notch processing in Drosophila Presenilin mutants.. Nature.

[19] Weng AP, Ferrando AA, Lee W, Morris JPt, Silverman LB (2004). Activating mutations of NOTCH1 in human T cell acute lymphoblastic leukemia.. Science.

[20] Aster JC, Pear WS, Blacklow SC (2008). Notch signaling in leukemia.. Annu Rev Pathol.

[21] Malecki MJ, Sanchez-Irizarry C, Mitchell JL, Histen G, Xu ML (2006). Leukemia-Associated Mutations within the NOTCH1 Heterodimerization Domain Fall into at Least Two Distinct Mechanistic Classes.. Mol Cell Biol.

[22] Harrison SC (2005). Mechanism of membrane fusion by viral envelope proteins.. Adv Virus Res.

[23] Willnow TE, Moehring JM, Inocencio NM, Moehring TJ, Herz J (1996). The low-density-lipoprotein receptor-related protein (LRP) is processed by furin in vivo and in vitro.. Biochem J.

[24] Ko KW, McLeod RS, Avramoglu RK, Nimpf J, FitzGerald DJ (1998). Mutation at the processing site of chicken low density lipoprotein receptor-related protein impairs efficient endoplasmic reticulum exit, but proteolytic cleavage is not essential for its endocytic functions.. J Biol Chem.

[25] Bush G, diSibio G, Miyamoto A, Denault JB, Leduc R (2001). Ligand-induced signaling in the absence of furin processing of Notch1.. Dev Biol.

[26] Nichols JT, Miyamoto A, Olsen SL, D'Souza B, Yao C (2007). DSL ligand endocytosis physically dissociates Notch1 heterodimers before activating proteolysis can occur.. J Cell Biol.

[27] Kidd S, Lieber T (2002). Furin cleavage is not a requirement for Drosophila Notch function.. Mech Dev.

[28] Li K, Li Y, Wu W, Gordon WR, Chang DW (2008). Modulation of Notch signaling by antibodies specific for the extracellular negative regulatory region of NOTCH3.. J Biol Chem.

[29] Ray WJ, Yao M, Mumm J, Schroeter EH, Saftig P (1999). Cell surface presenilin-1 participates in the gamma-secretase-like proteolysis of Notch.. J Biol Chem.

[30] Redmond L, Oh SR, Hicks C, Weinmaster G, Ghosh A (2000). Nuclear Notch1 signaling and the regulation of dendritic development.. Nat Neurosci.

[31] Peterson FC, Gordon NC, Gettins PG (2001). High-level bacterial expression and 15N-alanine-labeling of bovine trypsin. Application to the study of trypsin-inhibitor complexes and trypsinogen activation by NMR spectroscopy.. Biochemistry.

[32] Delaglio F, Grzesiek S, Vuister GW, Zhu G, Pfeifer J (1995). NMRPipe: a multidimensional spectral processing system based on UNIX pipes.. J Biomol NMR.

[33] Johnson BA (2004). Using NMRView to visualize and analyze the NMR spectra of macromolecules.. Methods Mol Biol.

[34] Aster JC, Robertson ES, Hasserjian RP, Turner JR, Kieff E (1997). Oncogenic forms of NOTCH1 lacking either the primary binding site for RBP-Jkappa or nuclear localization sequences retain the ability to associate with RBP-Jkappa and activate transcription.. J Biol Chem.

