# Supplementary figures and images for: Effects of S1 Cleavage on the Structure, Surface Export, and Signaling Activity of Human Notch1 and Notch2
Source: PLoS One. 2009 Aug 24;4(8):e6613. doi: 10.1371/journal.pone.0006613 (PMC2726630; doi:10.1371/journal.pone.0006613)

Figure S1

Figure S1-A

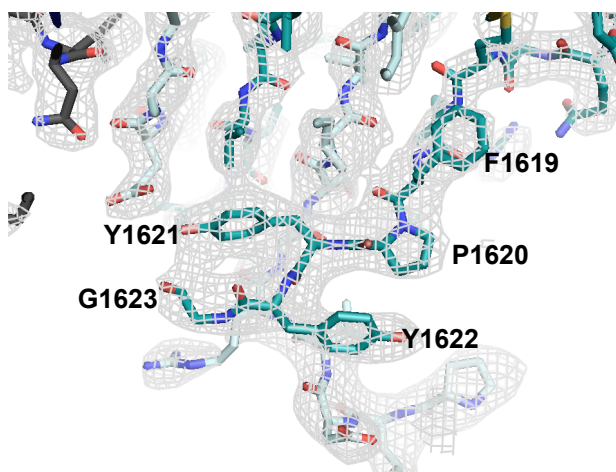

Figure S1-B

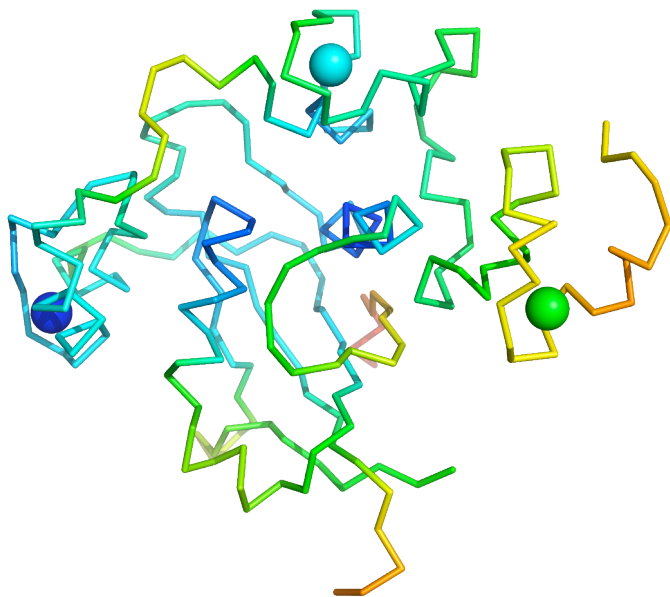

Supplement: Figure S1 — Electron density and ribbon diagram colored by B-factor. A. 2Fo-Fc electron density map of the furin loop region, contoured to a level of 1.2 σ. B. Ribbon diagram of the furin-cleaved N1-NRR colored by B-factor in Pymol in a continuum of colors from low B- factors (blue) to high B-factors (red). (0.95 MB PDF) [file pone.0006613.s001.pdf]

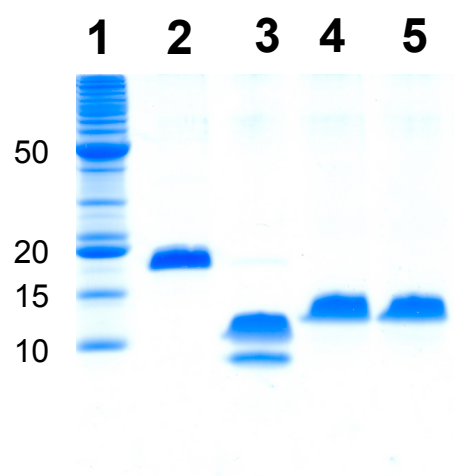

Figure S2

Supplement: Figure S2 — In vitro processing of the hN2 HD domain requires the S1 loop. (A) Normal human Notch2 HD (lanes 2 and 3) and human Notch2 HD-loopout (lanes 4 and 5) constructs before (lanes 2 and 4) and after (lanes 3 and 5) overnight cleavage at 37°C with recombinant furin. Lane 1 consists of molecular weight standards. (0.15 MB PDF) [file pone.0006613.s002.pdf]

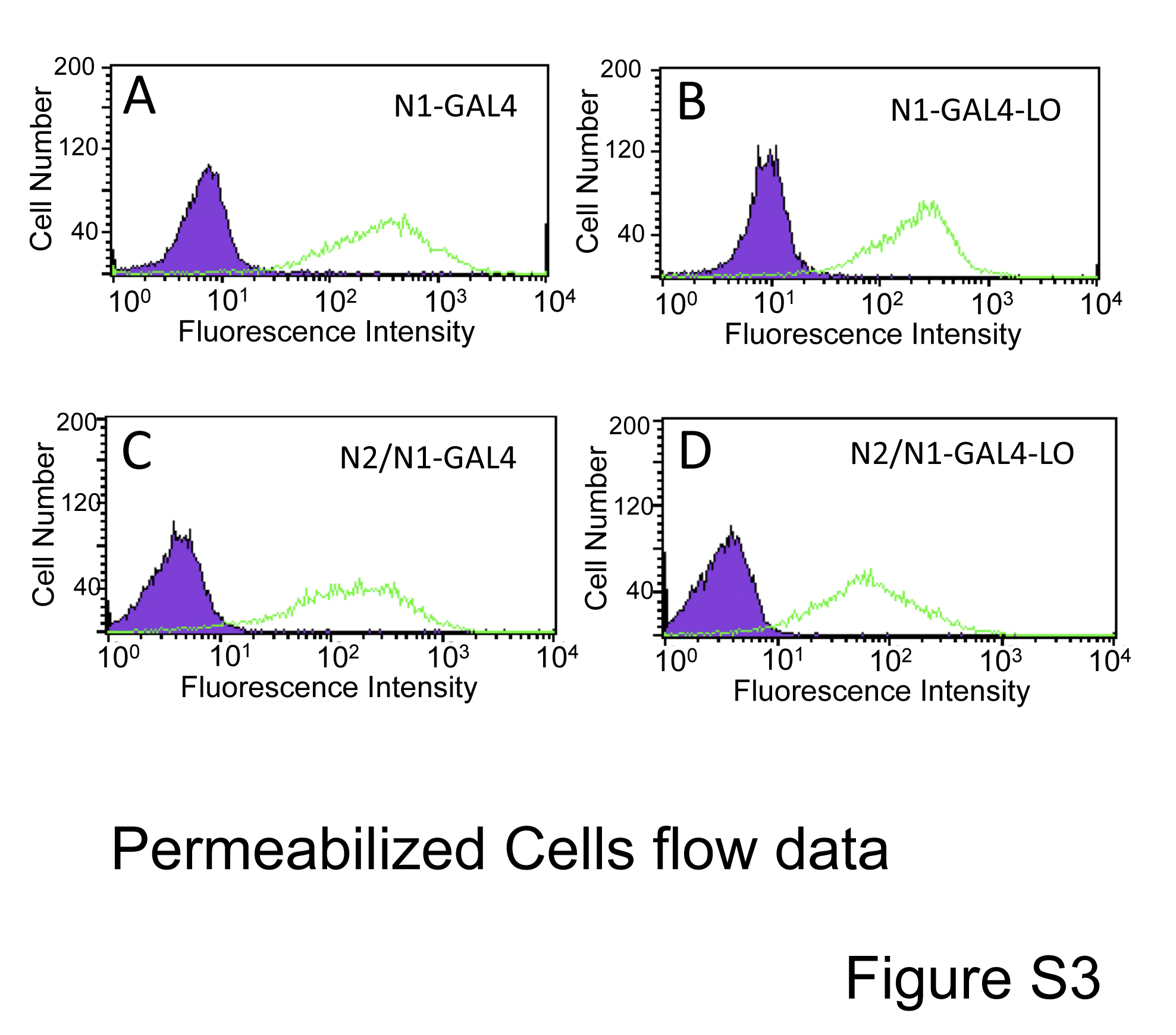

Supplement: Figure S3 — A-D. Flow cytometry of the indicated Notch-expressing cell lines after permeabilization. Receptors were detected with a FITC-conjugated anti-FLAG antibody (green). An isotype-matched antibody was used as a control (black and purple plots). (0.47 MB TIF) [file pone.0006613.s003.tif]

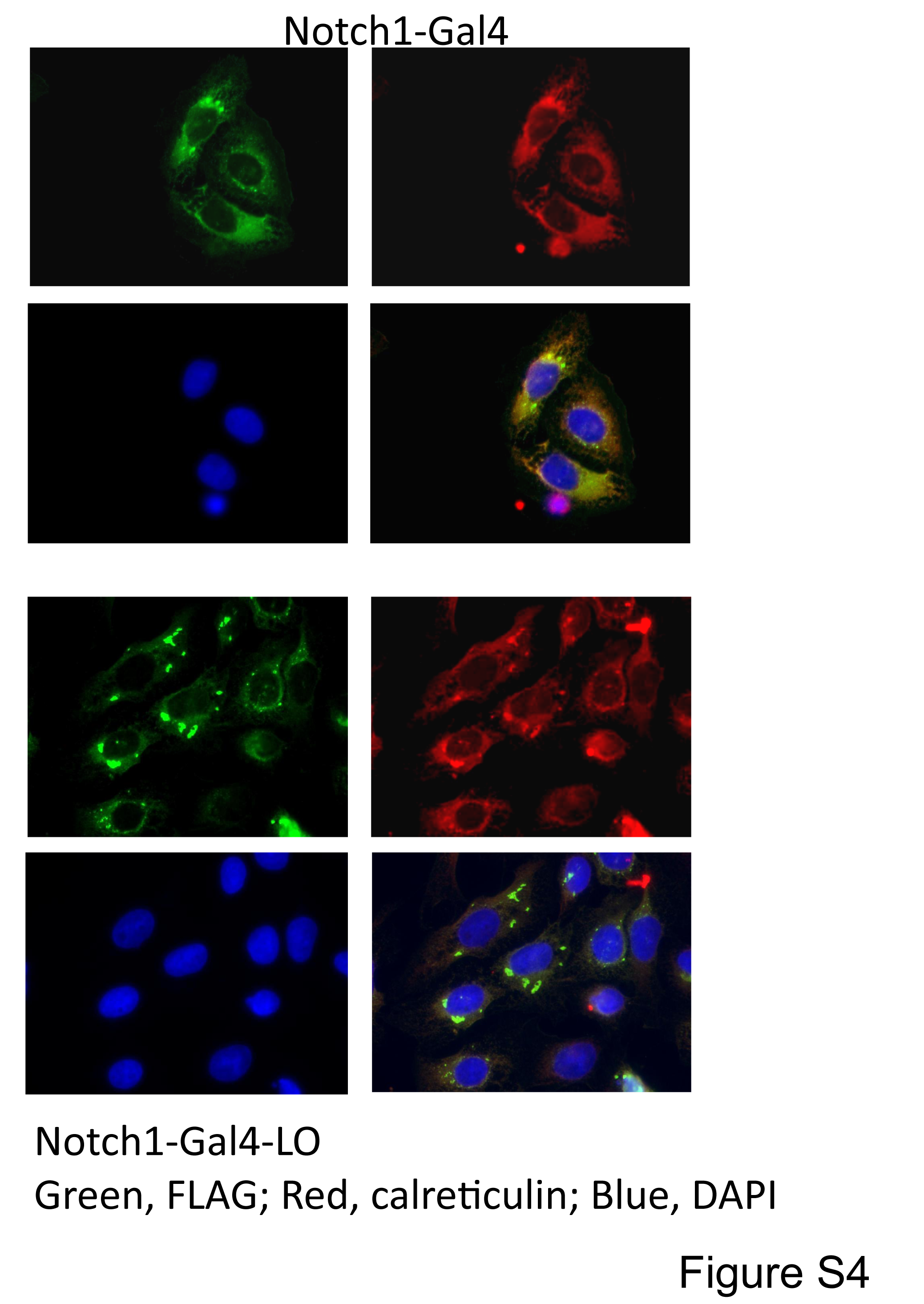

Supplement: Figure S4 — Immunofluorescent staining of U2OS cells stably transfected with Notch1-GAL4 (top) and Notch1-GAL4-LO (bottom) receptors. (1.67 MB TIF) [file pone.0006613.s004.tif]

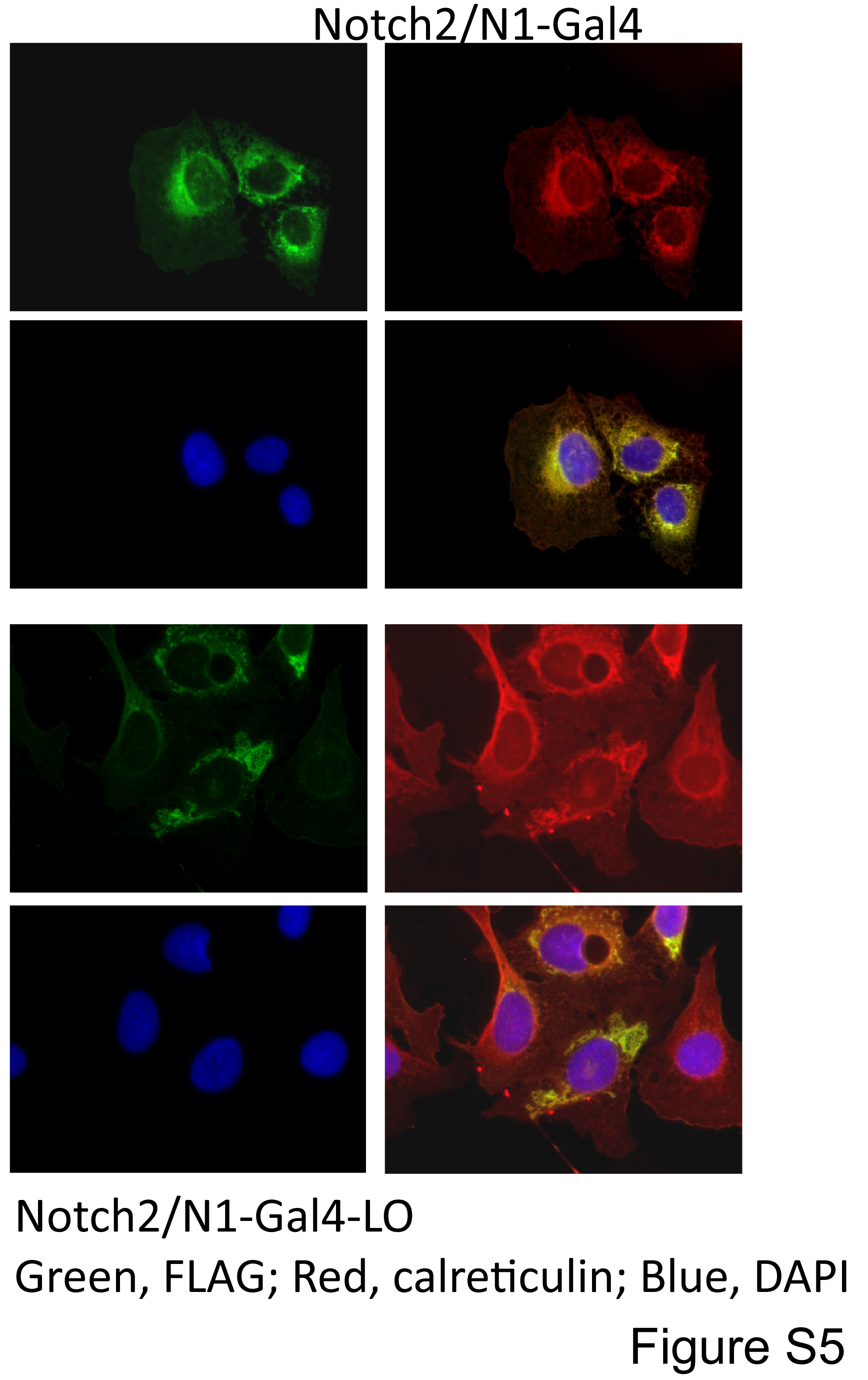

Supplement: Figure S5 — Immunofluorescent staining of U2OS cells stably transfected with Notch2/N1-GAL4 (top) and Notch2/N1-GAL4-LO (bottom) receptors. (1.62 MB TIF) [file pone.0006613.s005.tif]

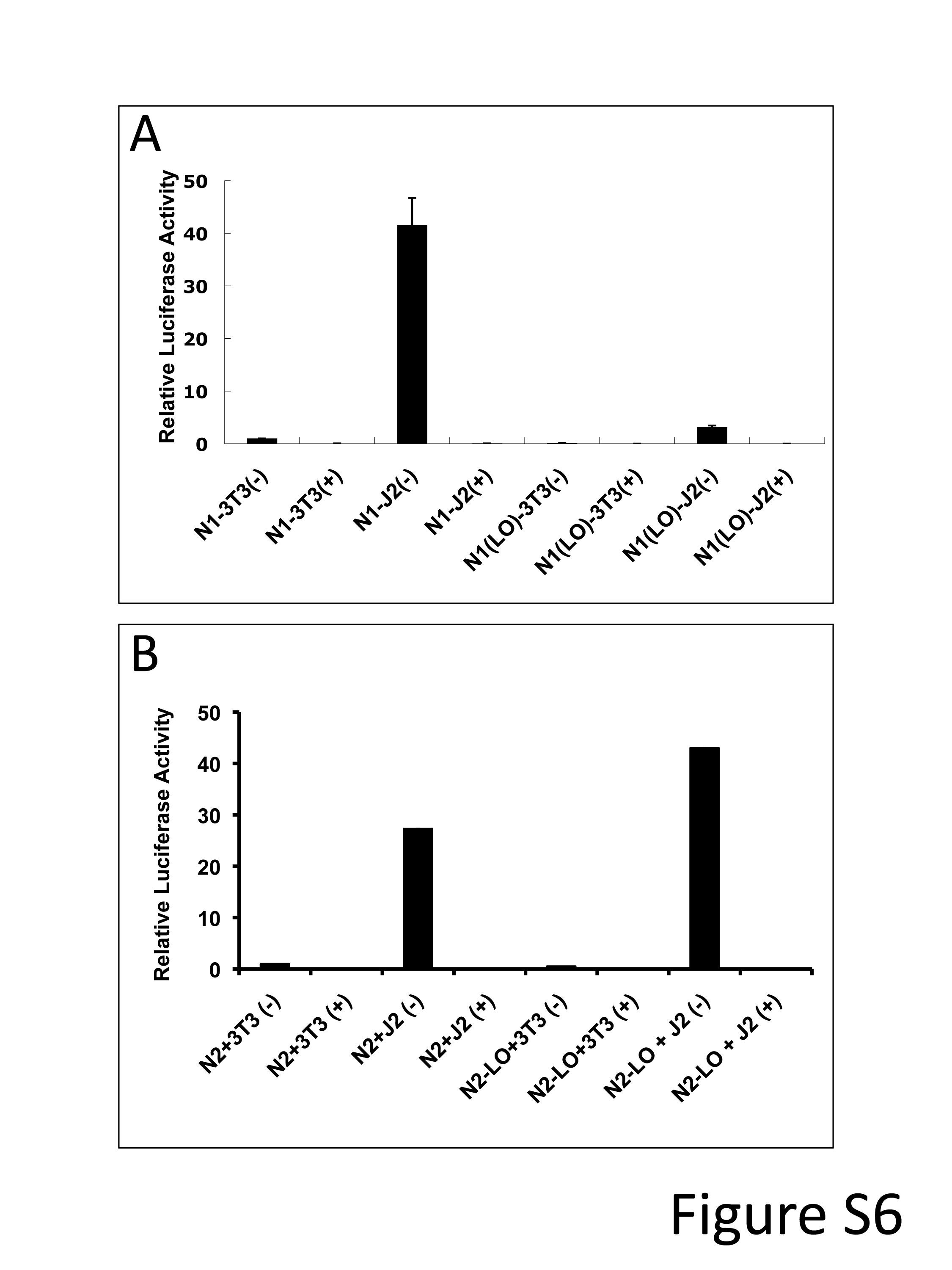

Supplement: Figure S6 — Activation of S1-cleavage-resistant Notch receptors is prevented by γ-secretase inhibitors. A. Chimeric Notch1 receptor activation was assessed in the presence (+) or absence (-) of the γ-secretase inhibitor compound E (1 µM). B. Chimeric Notch2 receptor activation was assessed in the presence (+) or absence (−) of the γ -secretase inhibitor compound E (1 µM). All data points were obtained in triplicate, with error bars indicating the standard deviation of the three replicates. Representative results from at least three independent experiments are shown. (0.22 MB TIF) [file pone.0006613.s006.tif]
